# The Abundance of Epicardial Adipose Tissue Surrounding Left Atrium Is Associated With the Occurrence of Stroke in Patients With Atrial Fibrillation

**DOI:** 10.1097/MD.0000000000003260

**Published:** 2016-04-08

**Authors:** Hsuan-Ming Tsao, Wei-Chih Hu, Ping-Huang Tsai, Chao-Lin Lee, Fang-Chun Liu, Hsueh-Han Wang, Li-Wei Lo, Shih-Lin Chang, Tze-Fan Chao, Shih-Ann Chen

**Affiliations:** From the Division of Cardiology, National Yang Ming University Hospital, Yi-Lan (H-MT) Department of Biomedical Engineering, Chung-Yuan Christian University, Taoyuan (W-CH); Division of Neurology, (P-HT, C-LL, F-CL); Department of Radiology, National Yang Ming University Hospital, Yi-Lan (H-HW); and Cardiovascular Research Center, Taipei Veterans General Hospital and National Yang Ming University, Taipei, Taiwan (L-WL, S-LC, T-FC, S-AC).

## Abstract

Epicardial adipose tissue (EAT) is positively associated with risk factors for cardiovascular disease, but the role of EAT in the development of atrial fibrillation (AF)-related stroke and its association with the anatomical and functional remodeling of the left atrium (LA) have not been elucidated.

This was a comparative cross-sectional study. Twenty-seven patients with paroxysmal or persistent AF and cardioembolic stroke were selected and compared with 68 age- and sex-matched AF patients without stroke. In addition, 20 controls without a history of AF or stroke were included. The periatrial EAT and the structural and functional properties of the LA and left ventricle were evaluated using contrast-enhanced 64-slice multidetector computed tomography during sinus rhythm. Total EAT around the LA was significantly increased across the groups (control vs AF vs AF-related stroke, *P* < 0.001). The volumes of the LA and the LA appendage (LAA) were also significantly increased across the 3 groups (*P* < 0.001 for each). The emptying fraction of the LA and LAA and the booster-pump function of the LA and LAA were all reduced across the 3 groups (*P* < 0.001 for all). In addition, the Hounsfield unit (HU) ratio of the LAA to the ascending aorta (LAA/AA) was also decreased in patients with stroke (*P* < 0.001). Furthermore, EAT had a negative correlation with the dynamic function of the LA, LAA, and the HU ratio. After a multivariate analysis, increased EAT (*P* < 0.001) was shown to be independently associated with the occurrence of AF-related stroke.

Periatrial EAT was increased and was correlated with atrial dysfunction in patients with AF-related stroke. Hence, EAT assessment may potentially offer an incremental value for grading the risk of cardioembolic stroke in patients with AF.

## INTRODUCTION

Epicardial adipose tissue (EAT) is the fat localized between the visceral pericardium and the myocardium. It acts as not only an anatomical fat depot but also a biologically active structure that can secrete many proinflammatory cytokines and adiponectin.^1–3^ The amount of EAT is reported to be associated with the development of coronary artery disease (CAD)^4–6^ and atrial fibrillation (AF).^7–9^ We have previously demonstrated that the EAT surrounding the left atrium (LA) is significantly increased in patients with AF and is related to the recurrence of AF after catheter ablation.^10^ It remains uncertain whether a specific compartment or the entire EAT of the heart contributes to the pathogenesis of AF.^11,12^

AF is well recognized as a significant risk factor for stroke. In addition, AF-related stroke is more likely to recur and results in more severe disabilities.^13,14^ Clinical scoring systems, including CHADS_2_ and CHADS_2_-Vasc scores, have been developed to stratify the risk of stroke and guide the appropriate antithrombotic therapy in patients with AF.^15,16^ However, improving the accuracy of stroke prediction is still an important task. Moreover, the anatomical and functional properties of the LA play a pivotal role in the development of AF.^17–19^ We hypothesize that the EAT located in the vicinity of the LA and the LA appendage (LAA) may be associated with the remodeling of the substrate and the subsequent development of AF-related stroke. This study was aimed at exploring the relationship between the amount of EAT and the dynamic function of the LA/LAA in relation to AF-related stroke using multidetector computed tomographic (MDCT) images.

## METHODS

### Study Population

This was an observational study with a comparative cross-sectional design. The patients with AF and a first episode of acute ischemic cerebral infarction who were admitted to our neurological ward were included for assessment. The cause of the stroke was classified as AF-related based on the definitions of the SSS-TOAST system.^20^ All stroke patients were diagnosed as having had an AF-related stroke if it was due to nonvalvular AF and no signs of other etiologies were present. The exclusion criteria included AF rhythm in the 48 hours after admission, serum creatinine >2 mg/dL, a history of contrast allergy, or inability of the participant to provide informed consent. The AF group included the AF patients, matched for age, sex, and AF type (paroxysmal/persistent), who had no history of stroke and who underwent MDCT before catheter ablation. In addition, the age- and sex-matched controls without AF or stroke who underwent MDCT for the evaluation of possible coronary diseases were included. This study was approved by the Institutional Review Board of National Yang Ming University Hospital.

### Computed Tomographic Studies

The hearts were evaluated with an ECG-gated, 64-slice MDCT scanner (Brilliance CT, 64-slice, Philip, Amsterdam, Netherlands). Participants underwent a CT scan during sinus rhythm. We administered 80 mL of the nonionic contrast medium iohexol (350 mg of iodine per milliliter [Optiray, Mallinckrodt, Canada]) followed by 20 mL of normal saline through the patient's antecubital vein using a power injector at a rate of 5.5 mL/s. We used the bolus tracing technique to monitor the signal intensity at the predefined region of interest (ROI) in the ascending aorta. When the CT number of the ROI reached the pre-set threshold (Hounsfield unit [HU]: 150), the scan automatically started and the patients were instructed to hold their breath during the acquisition of the images, which covered the area from the aortic arch to the cardiac apex (collimation 64 × 0.625 mm, gantry rotation time 400 ms, table speed 19 mm/s, tube voltage 120 kV, effective tube current 500–600 mA). The acquisition time was 12 to 14.5 s, depending on the heart rate.

### Image Analysis

**FIGURE 1:**
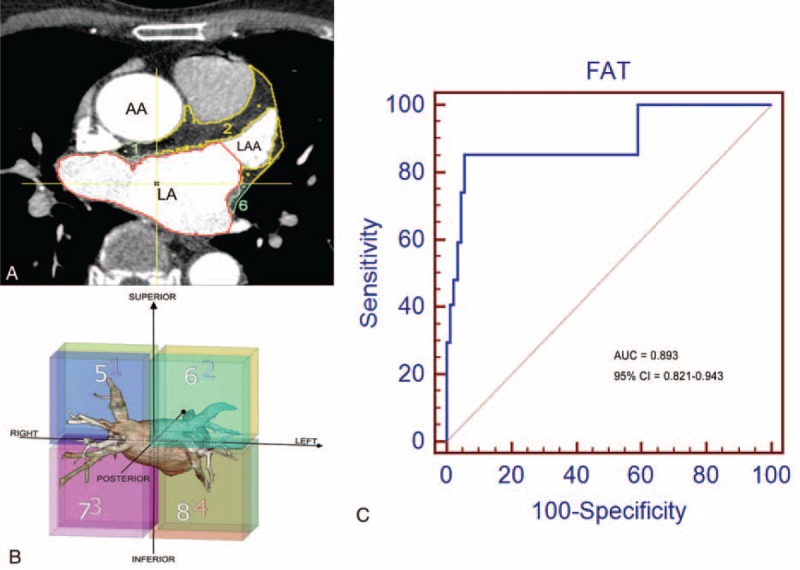
The method used to quantify the epicardial adipose tissue (EAT) surrounding the left atrium (LA). (A) EAT was identified using threshold attenuation values of −50 to −200 Hounsfield units (HUs) and the axial images at atrial end-diastole were used to trace the EAT from the pulmonary artery to the coronary sinus. (B) Eight zones around the LA were analyzed. (C) Receiver-operating characteristic (ROC) curve of the EAT amount to predict stroke in patients with atrial fibrillation. AUC = area under the curve.

All CT images were analyzed offline with software developed by the Department of Biomedical Engineering, Chung Yuan Christian University (Chung-Li, Taiwan).^21^ The axial images at atrial end-diastole were used to quantify the EAT surrounding the LA from the pulmonary artery to the coronary sinus. The volume of EAT was obtained using a semiautomated method, and the fat was recognized using threshold attenuation values of −50 to −200 HU. The investigator examined all images sequentially and adjusted the area of the EAT as necessary to assure the accurate inclusion of epicardial fat between the myocardium and the visceral pericardium. (Figure 1A, and see Video, Supplemental Video, which demonstrates the evaluation of epicardial fat around the LA). All images were verified for accuracy by 2 investigators. To understand the topographic distribution of the EAT, the periatrial space was equally divided into 8 regions. We divided the periatrial space equally along the X, Y, and Z planes. Zone 1 indicates the space of the right-anterior-superior LA, zone 2 indicates the space of the left-anterior-superior LA, zone 3 indicates the space of the right-anterior-inferior LA, zone 4 indicates the space of the left-anterior-inferior LA, zone 5 indicates the space of the right-posterior-superior LA, zone 6 indicates the space of the left-posterior-superior LA, zone 7 indicates the space of the right-posterior-inferior LA, and zone 8 indicates the space of the left-posterior-inferior LA (Figure 1B).

**FIGURE 2:**
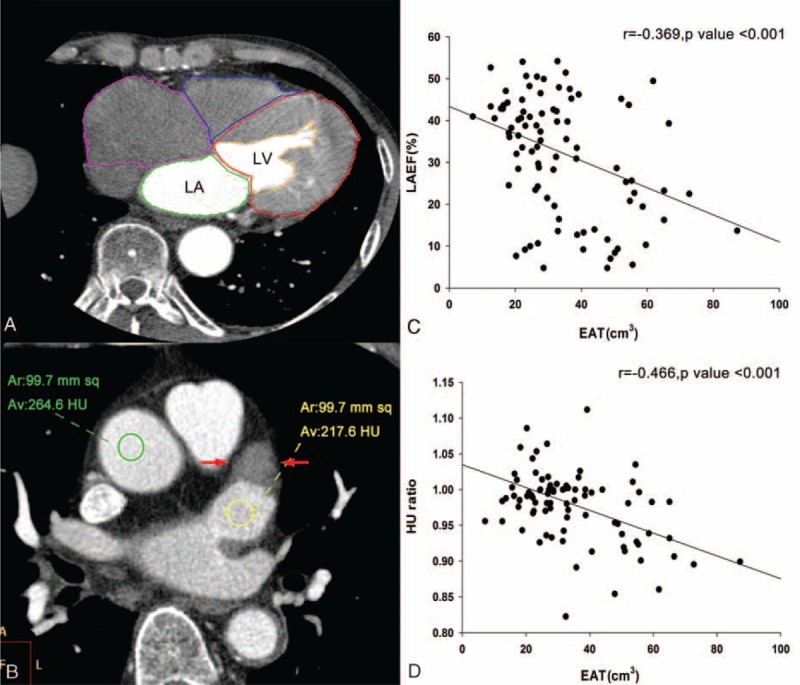
(A) The method used to evaluate left atrium (LA) and left ventricle function by tracing the endocardial border using multidetector computed tomographic images. (B) The method used to acquire the HU ratio of the LA appendage (LAA) and ascending aorta (AA). A 1-cm^2^ region of interest was sampled within the LAA to select the 1-cm^2^ region showing the lowest HU value. However, sampling within the filling defect of the LAA (between arrows) was avoided. At the same level, another 1-cm^2^ region was selected in the contrast-enhanced lumen of the AA. (C) LA emptying fraction (EF) was negatively correlated with the abundance of epicardial adipose tissue (EAT). (D) LAA/AA HU ratio was negatively correlated with the abundance of EAT.

The axial images were then reconstructed at multiple phases covering the cardiac cycle in increments of 10% of the R-R interval. Serial multiphase short-axis images were generated using semiautomated software to reformat the images to a slice thickness of 0.9 mm. The 10 serial images of LA and LAA were identified visually. The endocardial border was traced for each slice and, if necessary, manually adjusted to ensure accurate tracing (Figure 2A). A modified Simpson method was used to calculate the volume of the chambers at the 10 phases. The emptying fraction (EF) is defined as (maximal volume–minimal volume)/maximal volume. The active EF (booster-pump) = ([volume at P-wave beginning-minimal volume]/volume at P wave beginning) and the passive EF (conduit) = ([maximal volume − volume at P wave beginning]/maximal volume).

The circulation stasis of the LAA was evaluated based on the HU ratio of the LAA to the ascending aorta (LAA/AA) at the end-diastolic phase of the LAA, which was fully opacified by the contrast media. For quantitative analyses, a 1-cm^2^ ROI was sampled within the LAA to select the 1-cm^2^ region showing the lowest HU values outside the filling defect, if it existed. At the same level, another 1-cm^2^ region was selected in the contrast-enhanced lumen of the AA (Figure 2B). The HU values were measured independently by 2 investigators, and the mean values were used for the analysis.

### Statistical Analysis

Continuous variables with a normal distribution are presented as the mean ± standard deviation, and categorical variables are presented as absolute values and percentages. A *χ*^2^ test with Yates’ correction or Fisher exact test was used for the analysis of categorical data. A Student *t* test or the Mann–Whitney *U* test was used for the analysis of continuous data, as appropriate. Multivariate logistic regression was performed to identify the independent predictors of AF-related stroke. Inclusion criteria for multivariate analysis were set at *P* < 0.1 from the univariate analysis. A value of *P* < 0.05 was considered statistically significant. Data were analyzed using SPSS for Windows (version 21, SPSS Inc., Chicago, IL).

## RESULTS

### Patient Characteristics

**FIGURE 3:**
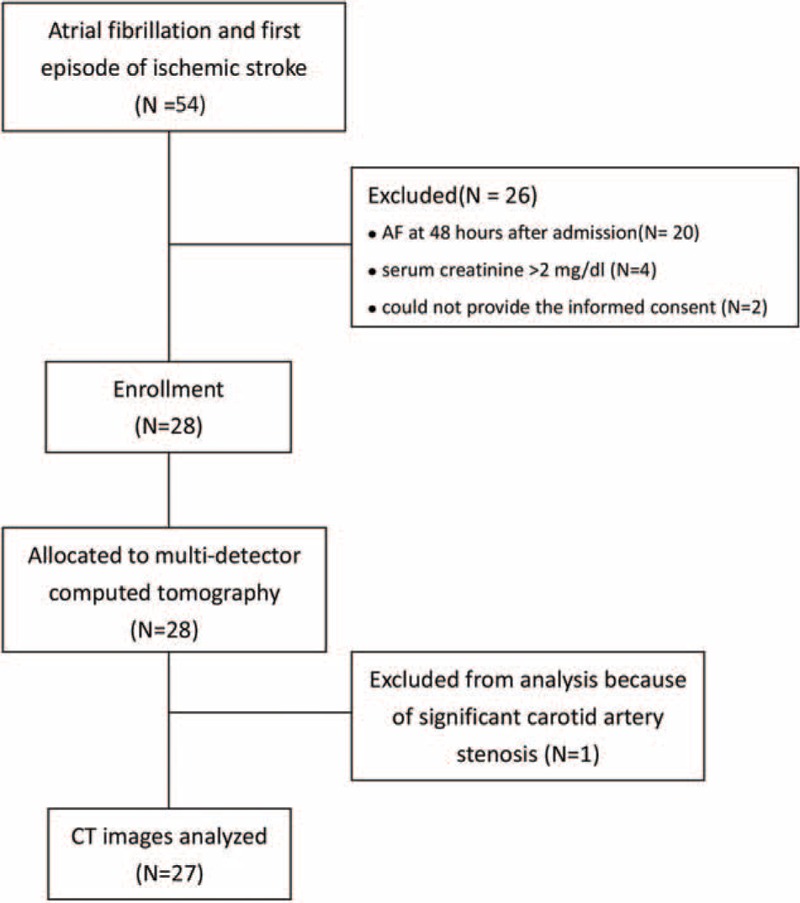
The flowchart of the participants’ progress in this study.

We analyzed the demographic, laboratory, and MDCT data that were collected from 27 consecutive patients with AF-related stroke between January 1, 2012 and February 28, 2013 who fulfilled the inclusion and exclusion criteria and provided informed consent (Figure 3). Sixty-eight AF patients without any history of stroke were selected for the comparison. In addition, 20 controls without a history of AF or stroke were included. The age, sex, body mass index (BMI), and prevalence of diabetes mellitus, coronary artery disease, and hypertension were similar among the 3 groups. Low-density lipoprotein cholesterol was not different among the 3 groups. The percentage of patients with paroxysmal AF and with CHADS_2_ scores <2 and CHADS_2_-Vasc scores <2 were similar between the AF patients with stroke and those without stroke. Furthermore, the percentage of patients using anticoagulant, antiplatelet, and statin medications was not significantly different (Table 1).

**TABLE 1:**
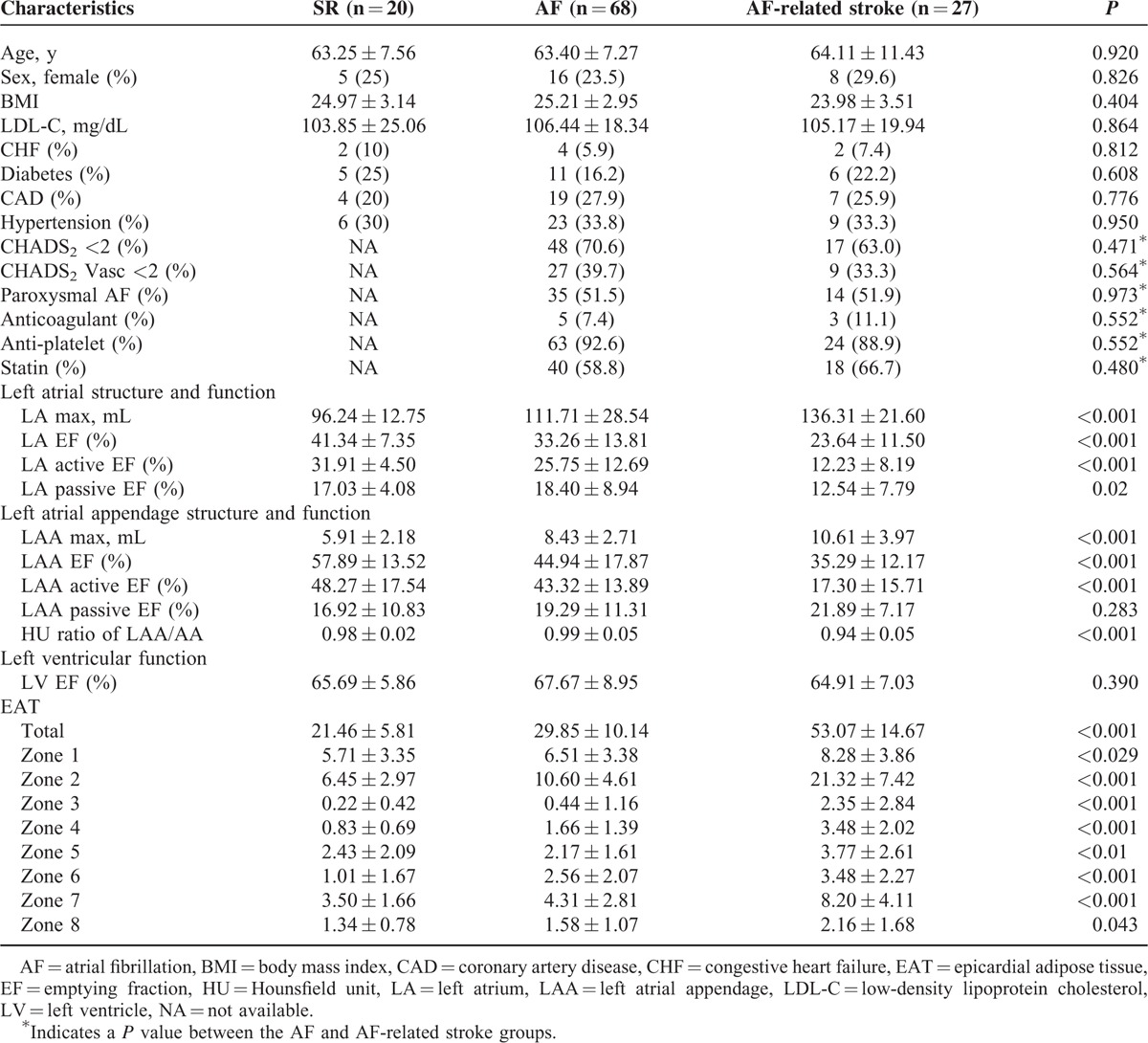
The Clinical and Computed Tomographic Characteristics of the Controls, Patients With Atrial Fibrillation and Patients With Atrial Fibrillation-related Stroke

### The Quantification and Distribution of Periatrial EAT

The total amount of periatrial EAT significantly increased across the patient groups from the control to AF to AF-related stroke groups (21.46 ± 5.81 vs 29.85 ± 10.14 vs 53.07 ± 14.67 cm^3^, respectively, *P* < 0.001). The topographic analysis showed that the EAT in every zone was significantly increased in patients with AF-related stroke (Table 1). The majority of EAT was accumulated in zones 1 and 2. The receiver-operating characteristic (ROC) curve analysis was performed to determine the cutoff value of total EAT for the prediction of AF-related stroke. The area under the curve (AUC) was found to be statistically significant (AUC = 0.893, 95% confidence interval = 0.821–0.943, *P* < 0.0001) (Figure 1C). As an optimal cutoff point, a high-risk EAT value of 40.68 cm^3^ was determined with an 85.2% sensitivity and 94.3% specificity.

We assessed the reproducibility of the EAT measurement technique in the first 20 patients with AF-related stroke and the 20 controls. There was good agreement in the intraobserver and interobserver measurements (*r* = 0.973 and 0.918, respectively).

### The LA/LAA Function and the Association With EAT

The EF (41.34% ± 7.35% vs 33.26% ± 13.81% vs 23.64% ± 11.50%, *P* < 0.001), active EF (31.91% ± 4.50% vs 25.75% ± 12.69% vs 12.23% ± 8.19%, *P* < 0.001) and passive EF (17.03% ± 4.08% vs 18.40% ± 8.94% vs 12.54% ± 7.79%, *P* = 0.02) of the LA (control, AF, and AF-related stroke, respectively) were significantly reduced across the patient groups. The EF (57.89% ± 13.52% vs 44.94% ± 17.87% vs 35.29% ± 12.17%, *P* < 0.001) and active EF (48.27% ± 17.54% vs 43.32% ± 13.89% vs 17.30% ± 15.71%, *P* < 0.001) of LAA (control, AF, and AF-related stroke, respectively) were also significantly reduced across the 3 groups. In addition, the total amount of periatrial EAT was negatively correlated with the EF (*r* = −0.369, *P* < 0.001) (Figure 2C), active EF (*r* = −0.326, *P* = 0.027) and passive EF (*r* = −0.286, *P* = 0.045) of the LA. In addition, the total EAT was negatively correlated with the active EF of the LAA (*r* = −0.464, *P* < 0.001).

### The HU Ratio of LAA/AA and the Association With EAT

The LAA/AA HU ratio was significantly decreased in patients with AF-related stroke compared with those without stroke (0.93% ± 0.05% vs 0.99% ± 0.06%, *P* < 0.001). In addition, the amount of periatrial EAT was negatively correlated with the LAA/AA HU ratio (*r* = −0.466, *P* < 0.001) (Figure 2D).

### The Severity of Stroke and the Association With EAT

The patients were categorized into 2 groups based on the severity of disability at 2 months after stroke. Group I included 16 patients with modified Rankin Scale scores of 0 to 2. Group II included 11 patients with modified Rankin Scale scores of 3 to 6. The amount of periatrial EAT was 53.43 ± 15.06 cm^3^ in group I and 52.56 ± 14.82 cm^3^ in group II (*P* = 0.88).

### Multivariate Analysis

To explore the independent predictors of AF-related stroke, a logistic regression analysis was performed to compare patients with AF-related stroke to those with AF without a stroke. Inclusion criteria for the multivariate analysis were set at *P* < 0.1 from the univariate analysis. Only total EAT (odds ratio [OR] = 1.12, *P* < 0.01) was independently associated with the development of stroke in patients with AF (Table 2).

**TABLE 2:**
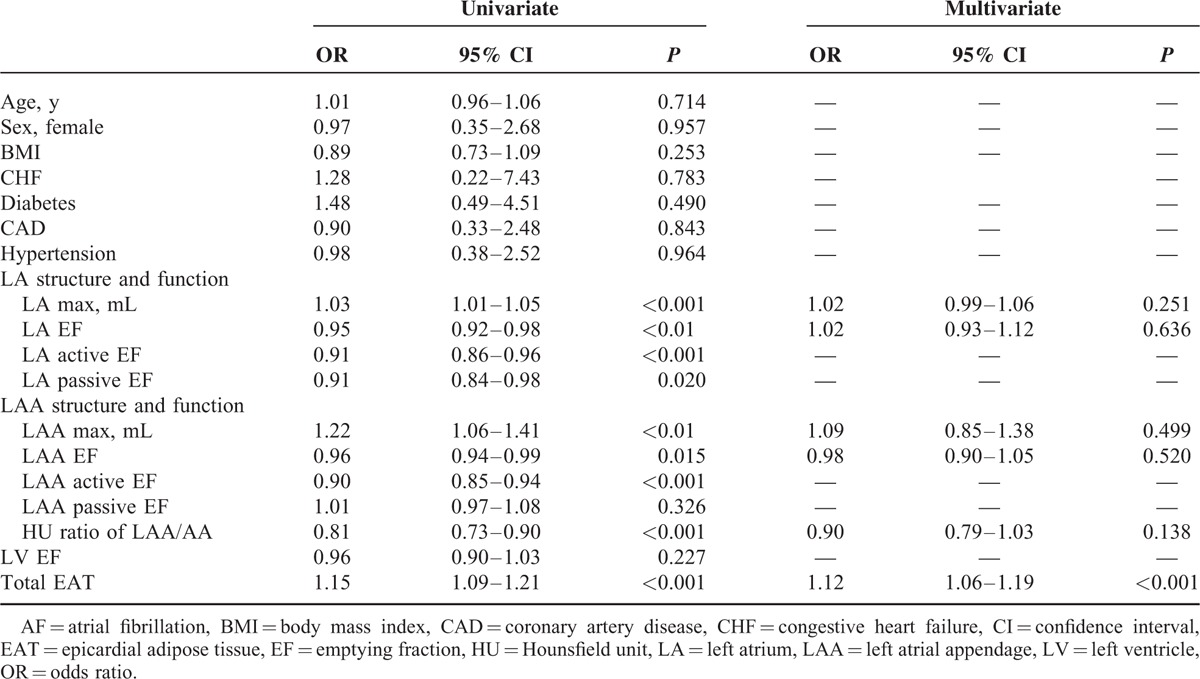
Logistic Regression Analysis for Predictors of Stroke in Patients With AF

## DISCUSSION

### Major Findings

Increased accumulation of EAT around the LA was independently associated with stroke in patients with AF. In addition, the abundance of EAT was negatively correlated with the mechanical function of the LA and LAA and also correlated with a decreased LAA/AA HU ratio. The contractile dysfunction of the LA/LAA ratio and the circulatory stasis of the LAA may account for the pathophysiology of EAT in patients with AF-related stroke.

### EAT and Stroke

Stroke is one of the most important threats to public health. Modification of the risk factors can prevent the development of stroke.^22,23^ Obesity and the clinical adiposity measures, including body mass index (BMI), waist circumference (WC), waist-to-hip ratio, waist-to-height ratio, and the ratio of subscapular to triceps skinfold thickness, have been demonstrated to have a positive relationship with ischemic stroke.^24–26^ Adipose tissue is believed to play an important role in the pathogenesis of stroke thorough the effects of powerful cytokines on the development of atherosclerosis, the renin angiotensin axis, and the activity of the sympathetic nervous system.^27^ However, the information regarding the association between EAT and stroke is minimal and controversial. CT assessment from the Framingham Heart Study of pericardial fat showed an insignificant association with stroke (OR = 1.29, *P* = 0.23) after adjusting for age, sex, BMI, and WC.^28^ However, Akil et al^29^ reported that the epicardial fat thickness measured at the free wall of the RV using echocardiography was significantly correlated with ischemic stroke. The present work, to the best of our knowledge, represents the first study trying to clarify the role of EAT in patients with AF and cardioembolic stroke using a comprehensive MDCT protocol.

### EAT and AF-related Stroke

Previous studies have underscored the importance of pericardial EAT in the development of AF, but the relationship between EAT and AF-related stroke has not been investigated. A CT evaluation from the Framingham Heart study showed that pericardial fat volume could predict AF risk independent of other measurements of adiposity.^7^ Al Chekakie et al^8^ demonstrated that pericardial fat volume is highly associated with paroxysmal and persistent AF independent of traditional risk factors. Batal et al^11^ reported that posterior LA fat thickness was positively linked to AF burden. We have also demonstrated that periatrial EAT was significantly increased in patients with AF and was associated with a higher incidence of AF recurrence after catheter ablation.^10^ In addition, serial research efforts have been dedicated to the exploration of the biological mechanisms that relate EAT and AF.^9,30–32^ These mechanisms include: the actions of proinflammatory cytokines and the adipo-fibrokines released from EAT, such as activin A, which can induce fibrotic changes of the atrial myocardium; adipocyte infiltration of the atrial myocardium, which can cause blockage of local conduction and promote the micro-reentry circuit; and potential modulations of the autonomic nervous system by the ganglionic plexus within the EAT, which may influence the occurrence of AF. In this study, we comprehensively assessed the relationship between the periatrial EAT, the functional properties of the LA, and the circulatory stasis of LAA with contrast-enhanced MDCT and found that the amount of EAT around the LA was independently associated with AF-related stroke, based on a multivariate analysis.

### Correlation of EAT With LA/LAA Function and Blood Stasis Within the LAA

The EF of the LA and LAA were significantly reduced in patients with AF-related stroke compared with the values of those without stroke. The active EF of the LA and LAA and the passive EF of the LA were also impaired. Furthermore, the LAA/AA HU ratio was decreased in patients with AF-related stroke. The LAA/AA HU ratio has been reliably used to identify LAA thrombus and to differentiate thrombus from spontaneous echo contrast (SEC).^33,34^ The CT attenuation value of the LAA can be reduced because of the lack of blood exchange with the LA blood pool and insufficient contrast enhancement. Therefore, the decreased LAA/AA HU ratio is attributed to the interplay between LAA dysfunction, the LVEF, and a prothrombotic state within the LAA and has been reported to be related to the degree of SEC.^35^ This may partly explain our finding that the reduced LA/LAA HU ratio was associated with the development of stroke in patients with AF.

In addition, both the dynamic function of the LA/LAA and the HU ratio were negatively correlated with the abundance of periatrial EAT. We speculate that the EAT plays an important role in the functional remodeling of the atrium and in thrombus formation in the LAA. Several laboratory reports have provided plausible reasons for this relationship.^36–38^ First, adipocytes can infiltrate into the atrial myocardium and result in the hypokinesia of the LA and LAA. Second, the adipocytokines secreted from EAT possess anticontractile properties and induce myocardial fibrosis. In addition, inflammatory cytokines may contribute to circulatory stasis and thrombus formation within the LAA. Our findings may shed the light on the pathogenesis of cardioembolic stroke because of AF. However, whether a therapeutic approach targeted on the pathophysiology of EAT can impact the development of AF-related stroke warrants further study.

### Clinical Implications

MDCT has been widely used for the delineation of LA anatomy and the collateral structures before catheter ablation of AF.^39^ In addition, coronary CT angiography also becomes a popular modality for the detection of CAD. Our findings justify the analysis of the EAT surrounding the LA instead of the analysis of the entire heart in AF patients. The quantification of EAT using MDCT can serve as an additional risk factor of AF-related stroke. Therefore, the abundance of periatrial EAT may potentially facilitate the guidance of appropriate antithrombotic therapy in low-risk patients with CHADS_2_ score <2.

## LIMITATIONS

Our study has several limitations. First, the number of patients with AF-related stroke was small because we only enrolled those with paroxysmal and persistent AF that could be converted to sinus rhythm. This was the only way to evaluate the triphasic LA function in detail. Therefore, this study's results may not be applicable to patients with permanent AF, which may represent 50% of all AF patients. Second, we acknowledge the possibility of confounding variables because of the concomitant use of medications. However, the prevalence of antithrombotic medication and statin use in AF stroke groups was similar. Third, we cannot avoid an inherent limitation related to the AF burden in a patient group that had no implanted devices. However, we only included those patients who were in sinus rhythm at 48 hours after admission. Lastly, we did not measure the adipocytokine levels in our patient groups, so the relationship between the CT findings and adipocytokines is speculative.

## CONCLUSION

The accumulation of EAT surrounding the LA is linked to the occurrence of stroke in our patients with paroxysmal and persistent AF. The reduced dynamic function of the LA and the circulatory stasis of the LAA were correlated with the abundance of EAT. Therefore, a quantitative assessment of periatrial EAT may provide incremental value, in addition to the assessment of the traditional risk factors, for the prediction of cardioembolic stroke in patients with AF. However, more basic and clinical studies are needed to better define the role of the EAT in AF-related stroke.
